# Strengthening the Clinical Practice Guideline Ecosystem

**DOI:** 10.1002/gin2.70051

**Published:** 2025-11-12

**Authors:** Nan Yang, Dong Roman Xu, Janne Estill, Yaolong Chen

**Affiliations:** ^1^ Evidence‐Based Medicine Center, School of Basic Medical Sciences Lanzhou University Lanzhou Gansu China; ^2^ Center for World Health Organisation Studies and Department of Health Management School of Health Management of Southern Medical University Guangzhou Guangdong China; ^3^ Southern Medical University Institute for Global Health (SIGHT) Dermatology Hospital of Southern Medical University (SMU) Guangzhou Guangdong China; ^4^ Acacia Lab for Implementation Science, School of Health Management and Dermatology Hospital Southern Medical University (SMU) Guangzhou China; ^5^ Institute of Global Health University of Geneva Geneva Switzerland; ^6^ Research Unit of Evidence‐Based Evaluation and Guidelines (2021RU017), Chinese Academy of Medical Sciences, School of Basic Medical Sciences Lanzhou University Lanzhou Gansu China; ^7^ WHO Collaborating Centre for Guideline Implementation and Knowledge Translation Lanzhou Gansu China

## Abstract

- A clinical practice guideline ecosystem framework is proposed, interlinking seven core elements of the guideline lifecycle into a dynamic, coordinated, and closed‐loop system.
- Guideline pre‐registration and protocol development are emphasized as crucial for enhancing transparency, efficiency, and avoiding duplication, yet remain underutilized in current practice.
- Guideline implementation and updating are critical to the ecosystem, requiring the integration of implementation science and novel approaches like artificial intelligence to overcome the “guideline bubble” and maintain currency.
- A call is made for establishing standardized frameworks to coordinate the elements and stakeholders within the ecosystem, systematically promoting the development, dissemination, and application of high‐quality guidelines.

The concept of clinical practice guideline has existed for over 30 years, but guidelines still face numerous limitations, including low methodological quality, inadequate reporting transparency, slow implementation, and infrequent updates [1, 2]. There are several barriers in the dynamics between the different steps of the entire process, ranging from guideline development to implementation for which improvements are warranted [3, 4]. The establishment of a cohesive and well‐coordinated guideline ecosystem could help to eliminate many barriers across the entire process from development to implementation and address the existing challenges.

Guidelines are one of the six main elements of the evidence ecosystem [5], interacting closely with the other elements. They are developed based on evidence generated and synthesised across various elements of the evidence ecosystem, with complex interrelationships between them [1]. We now place the guideline ecosystem within the broader context of the entire evidence ecosystem, which includes primary data collection, research, systematic reviews, and the identification of research gaps, forming a multifaceted, cyclical linkage [6, 7]. Both the guideline ecosystem and evidence ecosystem involve the similar stakeholders, including patients, the public, public health organisations, methodologists, policy‐makers and practitioners, and share the goal of improving healthcare practice [5]. Both ecosystems are dynamic, responsive to new evidence and practices, and require coordination to be effective [5]. By integrating these elements, we try to provide a more comprehensive view of the key elements within the guideline ecosystem (Figure 1):

1. *Review of existing guidelines:* Just as a systematic review of existing research should be conducted before undertaking any medical study [8]. While a full systematic review of existing guidelines is one approach, a focused search of reputable sources [9] (e.g., established guideline developer organisations) is often an efficient and legitimate strategy to (i) avoid duplication; (ii) facilitate a comprehensive understanding of existing recommendations; and (iii) provide primary sources, such as existing recommendations and evidence, for the adaptation of the guidelines [10].
2. *Preregistration and protocol:* Registering new guideline projects and developing protocols not only enhances transparency in the guideline development process, but also boosts efficiency and prevents duplication of efforts across guideline groups. The preregistration of guidelines started later than that of clinical trials and systematic reviews, but it has gained traction since dedicated platforms were established [11]. Currently, there are at least two platforms available for guideline developers globally to register their guidelines: the Guidelines International Network (GIN) library registry of guidelines, and the practice guideline registration transparency platform (PREPARE) [12, 13]. However, publishing protocol is still much less common for guidelines than for clinical trials or systematic reviews. To ensure scientific rigor, guideline protocols should outline the recruitment process and intended composition of the working group, management policy of conflicts of interest, methods of retrieval and evaluation of evidence, and consensus‐building procedures [14].
3. *Rigorous development:* The process of guideline development has been well‐documented and standardised by many guideline developer organisations and in the scientific literature. The key steps include the identification of clinical questions, evidence synthesis, quality evaluation, and the process of transforming the evidence into recommendations. Strict adherence to well‐established development guidance can strengthen the validity of the guidelines.
4. *Standardised and transparent reporting:* The Reporting Items for practice Guidelines in HealThcare (RIGHT) statement and its extensions provide instructions for writing different types of guidelines and different aspects of guidelines in a standardised manner [15]. Complete and clear reporting of the contents does not only improve understanding of the guideline among the users but also lays the foundation for a comprehensive assessment.
5. *Comprehensive evaluation:* Comprehensive evaluation should go beyond assessing the quality of the guidelines and include a focus on their uptake and the impact of the recommendations on health outcomes. While tools like the Appraisal of Guidelines for Research & Evaluation (AGREE) instrument and guideline implementability appraisal (GLIA) are commonly used to assess methodological quality and implementability [16, 17], comprehensive evaluation must ultimately focus on the impact of guidelines on health outcomes and clinical practice. Unevaluated guidelines may promote inappropriate and even harmful clinical practices [18, 19].
6. *Guideline implementation:* Contextual factors, such as guideline inflexibility and insufficient resources in the implementation setting, can hinder the implementation of otherwise well‐developed guidelines, leading to a ‘guideline bubble’. Collaboration between guideline developers and implementation scientists can improve the implementability of guidelines through guideline optimisation and reduce contextual barriers by implementation strategies. This, in turn, promotes the integration of guidelines into work routines [20]. However, implementation science has not yet garnered enough attention in many settings, particularly where resources are constrained.
7. *Updating guidelines:* Keeping guidelines up to date poses a significant challenge, prompting the emergence of the concept of ‘living guidelines’ [21]. However, maintaining guideline currency involves more than adopting a living approach; it requires a range of strategies, such as fixed‐interval updates, revisions triggered by significant new evidence, and continuous (living) modes of update. Artificial intelligence has the potential to improve the efficiency and cost‐effectiveness of updating guideline [22]. Protocols for guideline updates should be registered, completing cycle within the guideline ecosystem.

**Figure 1:**
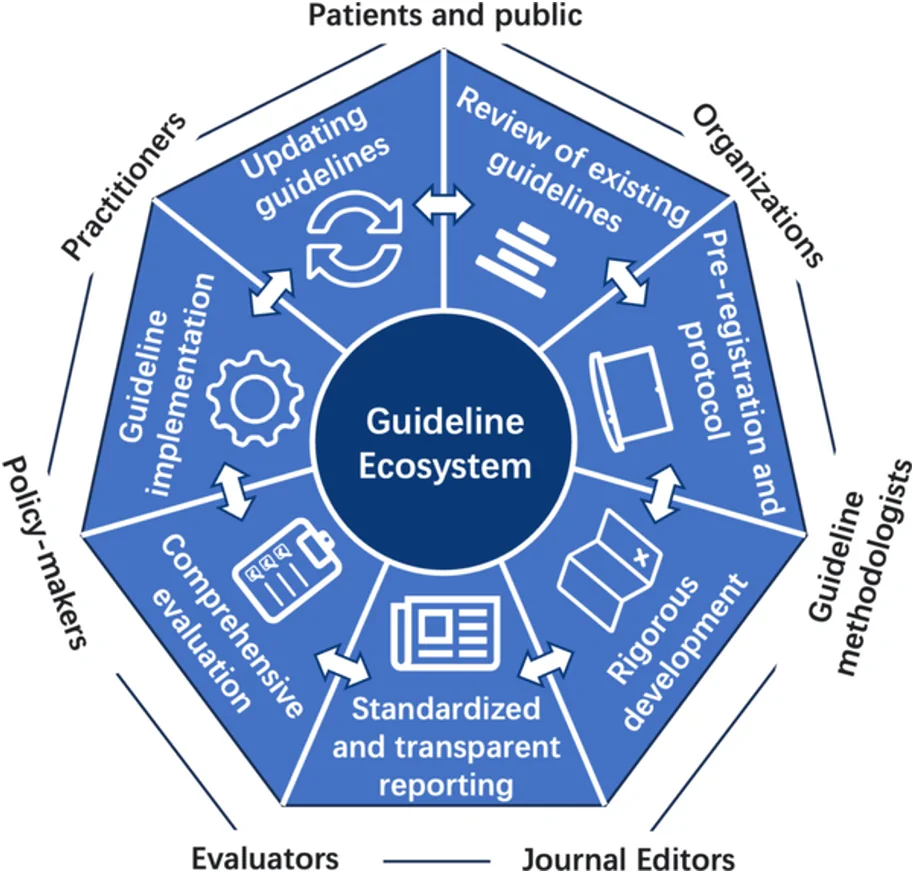
Visualises the conceptual framework that presents the guideline ecosystem, including key stakeholders and main activities from the review of existing guidelines through the various elements of the process to dynamic updates, completing the loop. The heptagon outlines the seven‐core elements that make this guideline ecosystem function. The ecosystem also requires an overarching infrastructure to coordinate and support stakeholders across the elements.

The guideline ecosystem encompasses more than the above steps, but we believe these seven are the most fundamental ones and deserve particular attention. Each of the steps will require a thorough evaluation of the current situation, as well as the development of a standardised framework. A standardised framework will outline the relationships between the different steps of the guideline ecosystem, which in turn will help to promote the development, implementation, and updating of high‐quality guidelines in a coordinated and transparent manner.

## Author Contributions

**Nan Yang:** conceptualization, writing — original draft, visualization. **Dong Roman Xu:** writing — review and editing, supervision **Janne Estill:** methodology, writing — review and editing. **Yaolong Chen:** conceptualization, writing — review and editing, supervision, funding acquisition. All authors have read and approved the final manuscript.

## Conflicts of Interest

The authors declare no conflict of interest. Yaolong Chen is the cofounder and cochair of RIGHT (Reporting Items for Practice Guidelines in Healthcare).

## Data Availability

All relevant data are within the manuscript.
